# Physical and functional cell-matrix uncoupling in a developing tissue under tension

**DOI:** 10.1242/dev.172577

**Published:** 2019-06-03

**Authors:** Amsha Proag, Bruno Monier, Magali Suzanne

**Affiliations:** LBCMCP, Centre de Biologie Intégrative (CBI), Université de Toulouse, CNRS, UPS, Toulouse 31062, France

**Keywords:** Extracellular matrix, Epithelium dynamics, Tension, Myosin, *Drosophila*, Developing leg

## Abstract

Tissue mechanics play a crucial role in organ development. They rely on the properties of cells and the extracellular matrix (ECM), but the relative physical contribution of cells and ECM to morphogenesis is poorly understood. Here, we have analyzed the behavior of the peripodial epithelium (PE) of the *Drosophila* leg disc in the light of the dynamics of its cellular and ECM components. The PE undergoes successive changes during leg development, including elongation, opening and removal to free the leg. During elongation, we found that the ECM and cell layer are progressively uncoupled. Concomitantly, the tension, mainly borne by the ECM at first, builds up in the cell monolayer. Then, each layer of the peripodial epithelium is removed by an independent mechanism: while the ECM layer withdraws following local proteolysis, cellular monolayer withdrawal is independent of ECM degradation and is driven by myosin II-dependent contraction. These results reveal a surprising physical and functional cell-matrix uncoupling in a monolayer epithelium under tension during development.

This article has an associated ‘The people behind the papers’ interview.

## Abstract (precis)

**Highlighted Article:** The independent behavior of extracellular matrix and cell monolayer of an epithelium under tension during *Drosophila* leg development highlights the interplay between tissue mechanics and cell-matrix coupling.

## INTRODUCTION

For many years, research has focused on the genetic and biochemical regulation of developmental processes. More recently, the development of new approaches based on live imaging and micromanipulation has brought novel insight into the physical properties of cells and tissues during morphogenesis, demonstrating the importance of cell and tissue mechanics during development. In particular, tissue-endogenous mechanical forces play an important role in tissue remodeling processes such as tissue elongation or folding (Bertet et al., 2004; Blankenship et al., 2006; Fernandez-Gonzalez and Zallen, 2011; Monier et al., 2015; Heisenberg and Bellaïche, 2013). Tissues have viscoelastic properties that depend for the most part on the architecture and dynamics of both cytoskeletal networks and extracellular matrix (ECM) (Ingber, 2006). On one hand, the extracellular matrix, which consists of a meshwork of multiple components, including collagen IV, laminins and perlecan, provides support to the epithelium (Theocharis et al., 2016; Miller, 2017). On the other hand, cell contractility relies mainly on the activity of acto-myosin, a macromolecular machinery composed of self-assembled actin filaments and non-muscle myosin II (Lecuit et al., 2011).

The role of cell mechanics in tissue deformation is beginning to be well characterized at a local scale, over relatively short periods of time and considering the epithelial sheet as a single viscoelastic entity. However, characterizing the respective role of the ECM and the cell monolayer in global tissue mechanics over long timescales remains difficult due to technical limitations. A key issue in the field is therefore understanding how the mechanical properties of cells and the extracellular matrix integrate to confer its physical properties to the tissue in living organisms (Daley and Yamada, 2013).

**Fig. 1.**
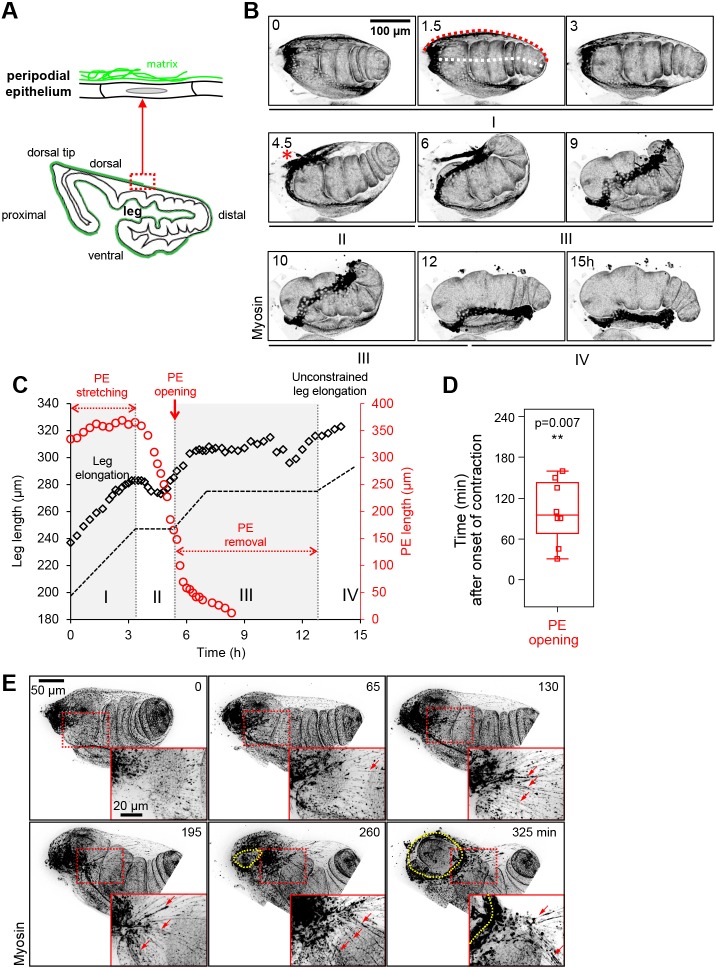
**Lengthening of the PE and myosin redistribution during leg elongation.** (A) Scheme of the leg disc at the start of elongation. The leg proper is surrounded by the peripodial epithelium (PE), which is composed of a thin squamous monolayer lying on a basement membrane. The basement membrane (green) is located on the outer side of the PE. (B) Leg disc eversion in culture. Time-lapse confocal microscopy images (*z*-projections) of a leg disc expressing fluorescent myosin (*sqh::sqhGFP*). A red asterisk indicates rupture at the dorsal tip (see also Movie 1). *n*=100 leg discs. Red and white dotted lines at time point 1.5 h denote, respectively, the length of the PE and of the leg. Roman numerals denote phases described in the text. (C) Dynamics of leg and PE elongation, showing the length of the leg (black) shown in B and of its PE (red) over time. The black dashed line is a simplified view of leg elongation dynamics. The dimensions measured on the tissue are indicated in B (time point 1.5 h). Phases I-IV are described in the text. The transition from phase I to II corresponds to the end of leg elongation, from phase II to III to the opening of the PE and from III to IV to the complete eversion. (D) Occurrence times of PE opening with respect to the onset of PE contraction. Boxes show median and quartiles, and whiskers encompass all values (eight leg discs). Opening occurs after contraction, as assessed by the one-sided Wilcoxon signed-rank test between the absolute time values paired per leg disc. (E) Time-lapse confocal images (*z*-projection) of the opening site in a leg disc (dorsal view) expressing fluorescent myosin (*sqh-TagRFPt[9B]*) and showing the formation of myosin cables (red arrows) radially distributed just distally from the opening site (yellow outline). *n*=9 leg discs. Insets indicate enlarged views of the dotted red rectangle (see also Movie 2).

To characterize the respective contribution of cells and the matrix in tissue morphogenesis, we took advantage of the isolated developmental system that is the *Drosophila* imaginal leg disc. The *Drosophila* leg disc is composed of two juxtaposed tissues: the peripodial epithelium and the leg proper. The peripodial epithelium (PE) surrounds the developing leg, and both tissues are joined together at the proximal region of the disc (Fig. 1A). As for any epithelia, the PE is composed of a cell monolayer and an underlying ECM called the basement membrane. Furthermore, at prepupal stage, cell division is mostly absent (McClure and Schubiger, 2005) and the pool of extracellular matrix components, produced by the fat body and the hemocytes, which are not present in the culture, is most probably not renewed (Fessler and Fessler, 1989). Thus, with a given number of cells and a given amount of matrix, the leg disc constitutes a relatively simple model system for addressing the contribution of mechanics in a developing tissue. Interestingly, the basement membrane forms the outermost layer of the leg disc with the cell monolayer lying right underneath and constituting a very thin squamous epithelium. This configuration makes the PE easily accessible to micromanipulation. Furthermore, imaginal discs develop normally in culture (Fristrom and Fristrom, 1993; Aldaz et al., 2010, 2013), indicating that they behave as independent entities whose mechanics can be characterized throughout the whole eversion process. In the wing disc, it has been shown that this stage is controlled by the downregulation of intercellular junctions, epithelial-mesenchymal transition (Pastor-Pareja et al., 2004; Manhire-Heath et al., 2013) and matrix metalloproteinase (MMP)-dependent ECM proteolysis (Srivastava et al., 2007), features that are specific to the dorsal tip where the PE opens. Furthermore, myosin accumulates in the PE in the wing disc and participates in PE opening and removal (Aldaz et al., 2013). However, the respective contribution of both layers was not addressed in this system.

Using the leg disc as a model, we investigate the dynamics of a tissue under tension and identify specific contributions of the cell monolayer and the ECM of the PE during leg development. We focus rather on the PE as a whole and show that the PE cell monolayer behaves independently from its basement membrane in terms of tension, opening and withdrawal. The PE is subjected to tension, which is initially mostly borne by the ECM. However, we find that the ECM gradually detaches from the cell monolayer and tension increases in the monolayer. In a second stage, the PE opens and retracts to allow leg eversion. Here, we show that the opening of cell monolayer is independent of ECM degradation and reveal that, in addition to the biochemical signal of MMP production, another independent step is required for PE removal. This highlights a combination of biochemical and mechanical signals: local ECM proteolysis and myosin II-dependent monolayer withdrawal. Thus, these data reveal physical and functional cell-matrix uncoupling during a developmental process.

## RESULTS

### Peripodial epithelium expansion and removal during leg elongation

During metamorphosis, leg discs evert, going from a flat to a tubular elongated structure that prefigures the adult leg (Fristrom and Fristrom, 1993; von Kalm et al., 1995; Milner, 1977). We performed time-lapse fluorescence microscopy on leg discs cultured in the presence of ecdysone to follow the global dynamics of the PE during leg evagination. We discovered that the process is very stereotyped and proceeds through the following steps (Fig. 1B,C and Movie 1). First (phase I), the leg elongates, a process that relies on cell shape changes and rearrangements (Condic et al., 1991). While the leg elongates at constant speed, the PE gradually lengthens (*n*=72/72), until leg elongation slows down to a plateau (*n*=19/19) and PE length reaches its maximum. During phase II, leg elongation is strongly reduced and the PE shortens, (*n*=19/19). Interestingly, this often coincides with leg bending dorsally (*n*=56/94). Then the PE opens at the dorsal tip and starts to retract (phase III) (*n*=94/94). During PE retraction, the leg straightens. Leg elongation remains negligible while the PE retracts from dorsal to ventral. Last, the PE is totally removed and leg elongation resumes (phase IV).

These observations strongly suggest that, as in the wing (where PE contraction induces folding of the disc proper) (Aldaz et al., 2013), there is constant mechanical interplay between the leg and the PE. Indeed, leg elongation coincides with PE elongation and both movements slow down at the same time, suggesting that leg elongation could extend the PE to a maximal length. At this point, the leg halts, and frequently bends dorsally in the direction of the future opening site, suggesting that bending may be due to PE contraction. Finally, once the PE is totally removed, the leg elongates again, most probably free from external constraint.

### Active withdrawal of the PE by myosin-dependent contraction

As PE expansion reaches a maximum before the time of PE opening, we hypothesized that the momentary slowdown of leg elongation (Phase II) might indicate that the PE is under maximum strain, preventing further elongation of the leg. We decided to decipher first the physical properties of the PE during its elongation (phase I) and characterize myosin distribution and tension pattern.

In the leg PE, we found that myosin II is essentially cytoplasmic in early phase I, with essentially small dot-like accumulations and rare short linear enrichments (Fig. 1E). As the PE elongated, we could observe the formation of linear myosin structures in the dorsal region of the PE. Myosin reorganized into those cable-like structures extended radially from the future opening site at stage II (Fig. 1E and Movie 2, *n*=9/9). Finally, the PE contracts, which systematically preceded PE opening (Fig. 1D, *n*=8/8).

**Fig. 2.**
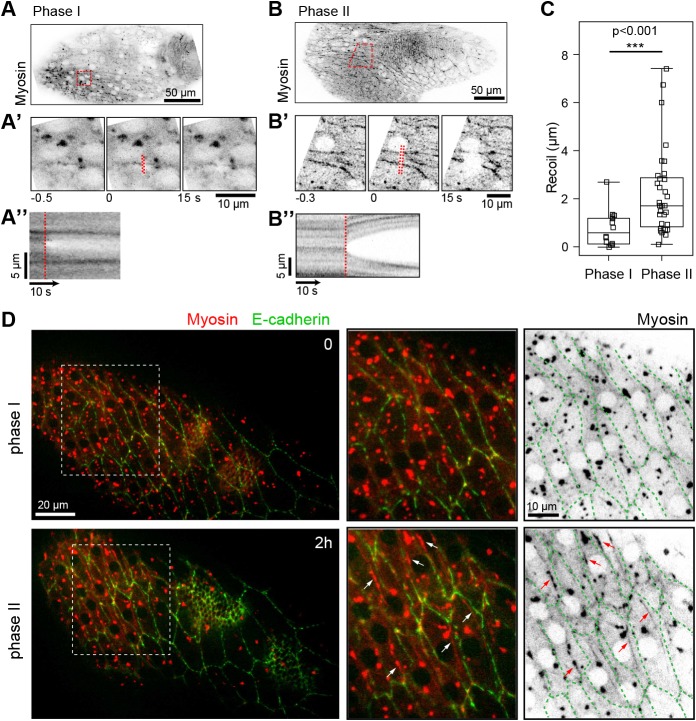
**Tension increases in the PE monolayer during leg elongation.** (A-C) Laser dissection of myosin filaments in the proximal dorsal region of the PE during phase I (A) or II (B). Phase I corresponds to 0-1 h APF; phase II to 2-3 h APF. Discs were maintained in culture for no more than 1 h and we made sure that PE opening had not occurred before performing experiments. (A,B) *Z*-projections of a leg disc expressing fluorescent myosin (*sqh-eGFP[29B*]). (A′,B′) Time-lapse confocal images (single plane) of the region framed in red in A,B. Red dotted lines indicate laser illumination. (A″,B″) Kymograph along the filament shows recoil dynamics (red dotted lines denote the illumination time point). (C) Recoil distance after laser dissection. Boxes show median and quartiles, and whiskers encompass all values. Phase I, *n*=14 leg discs (0-1 h APF); phase II, *n*=32 leg discs (2-3 h APF). Statistical significance was assessed using the one-sided Mann–Whitney test. (D) Time-lapse confocal images of a leg disc expressing fluorescent myosin (*sqh-TagRFPt[9B]*) and E-cadherin (*E-Cad-KI[GFP]*). Whereas myosin initially forms dot-shaped structures (phase I), it becomes enriched into cable-like structures in phase II (arrows), which appear uncoupled from the adhesion belt formed by adherens junctions. *n*=14 leg discs. In the rightmost panels, adherens junctions are outlined based on E-cadherin localization.

The drastic reorganization of myosin II prior to PE opening suggests important mechanical modifications. To further characterize the PE contractile state, we sought to measure mechanical tension in the PE at different stages of the elongation process using laser dissection. The highly anisotropic shape of these cells from the very start of the elongation process, together with the near absence of cell division and cell intercalation, suggested that tension might already be important at early phase I (McClure and Schubiger, 2005). Surprisingly, however, we observed negligible recoil either at junctions or at the cytoskeleton level in peripodial cells (Fig. 2A-A″,C; *n*=14 and Fig. S3A,C). The same laser power led to significant recoil in the cells of the leg proper, which was indicative of efficient laser cutting (Fig. S3A,B). Later on, however, the mechanical properties of the PE change drastically as laser dissection of myosin cable-like structures elicits higher recoil during phase II (Fig. 2B-B″,C, *n*=32), although the recoil at junctions appears to be very low (Fig. S3B,C), supporting the idea that tension is not borne by the adhesion belt in this epithelium. Indeed, the spatial organization of the myosin network does not colocalize with the adhesion belt (Fig. 2D). Given that the myosin content of cytoskeletal fibers increases from phase I to phase II (Fig. 1E), the higher recoil observed in phase II indicates higher tension in the actin-myosin fiber (see Materials and Methods). Hence, tension was greatly increased during PE elongation, starting very low at phase I and much increased at phase II, i.e. the end of leg elongation. This strongly suggests that the PE may become stretched as the leg elongates, and that the monolayer may reach a state of maximal tension when PE expansion is maximal at the end of leg elongation, before opening takes place.

As PE contraction systematically preceded PE opening, we asked whether contraction could lead to both events. We then attempted to inhibit PE contraction by targeting myosin II. Strong myosin II inhibition in the PE (achieved by expressing dominant-negative forms or by RNAi) tended to alter epithelial integrity. Therefore, we set up conditions in which contraction was visibly affected without notably perturbing the general organization of the tissue. Under these conditions, we observed that PE removal is prevented, while opening appears to take place normally (Fig. S2, *n*=7/7). Thus, myosin II organizes into dense fibers in the center of the dorsal region of the PE in the leg disc, and is required for PE removal. We could not conclude, however, on a role for myosin in PE opening as the absence of any opening defect could be due to the mild inhibition of myosin II in these conditions.

### Reorganization of the basement membrane during PE elongation

Although high tension in the PE is consistent with the reorganization of myosin II culminating during phase II, it was unexpected that the PE showed little tension at early phase I. A particular aspect of the PE is its thinness (around 1-2 μm): as it is composed of a thin cell monolayer lying on ECM, this matrix layer may help resist tension. To test this hypothesis, we investigated whether the ECM layer could contribute to storing the tension produced in the PE at the initiation of PE elongation.

**Fig. 3.**
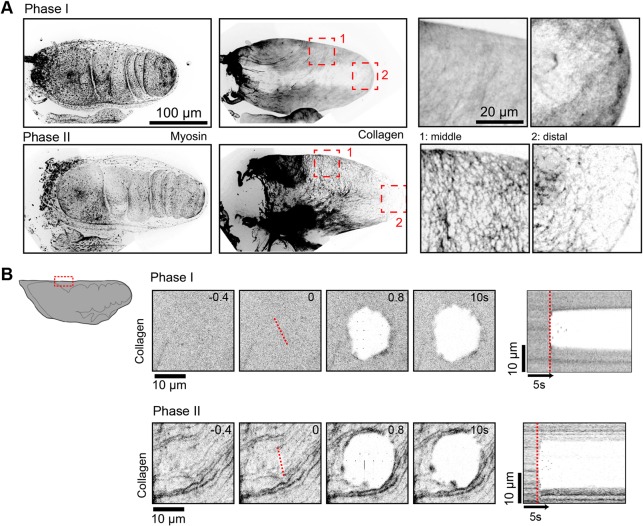
**Tension borne by the ECM layer during PE stretching.** (A) Confocal images (*z*-projections) of a leg disc (dorsal view) expressing fluorescent collagen (*vkg-GFP*) and myosin (*sqh-TagRFPt[9B])*, in phase I (upper panels) and phase II (lower panels). Right panels: enlarged views of the regions enclosed in red dashed squares, at the middle of the proximal-distal axis (1) and at the distal region (2). Contrast is increased in the enlarged view of region 2. *n*=19 leg discs. (B) The ECM layer is under tension during phases I and II. Left: laser dissection of the ECM layer of the PE of a leg disc expressing fluorescent collagen (*vkg-GFP*). The ECM layer was illuminated in a straight line (dotted red) at time point 0, leading to instant retraction of both sides of the cut. Right: kymographs of the recoil dynamics. *n*=25 leg discs in phase I and 8 in phase II. Average initial recoil distance is 8.1±5 µm (mean±s.d.).

As ECM is most probably not renewed during leg elongation, we first asked whether the ECM was remodeled as the PE elongated. To characterize the dynamics of ECM, we imaged fluorescent matrix components (collagen IV and perlecan) and observed that the ECM network underwent extensive remodeling between Phase I and II. From an initially continuous distribution along the proximal-distal axis, the matrix became sparser as elongation progressed (Fig. 3A, *n*=19/19). High-resolution images revealed gaps between fibers in the matrix layer (Fig. 3A, right panels), especially at the distal pole, where 20 µm large holes could be seen.

Then, to assess whether the ECM layer is subjected to mechanical strain, we performed laser dissection on the matrix layer. Cutting the matrix either in phase I or phase II led to immediate retraction (Fig. 3B, *n*=25/25 in phase I and 8/8 in phase II), preventing any comparison between the two developmental stages. However, it indicates that the matrix bears high tension all along the process of leg elongation. Together with previous experiments indicating that very low tension is borne by the cell monolayer, it indicates that while the tissue is under tension from the beginning of prepupal stage (start of phase I), the tension is borne mostly by the ECM.

These results show that the PE basement membrane initially stores most of the tension present in this stretched tissue, allowing the PE cell monolayer to remain in a relaxed state. But as the basement membrane gradually deteriorates, this loss of integrity must translate into a decrease in mechanical resistance to tension at the end of elongation, which may subject the PE cell monolayer to a larger fraction of the tension exerted by the elongating leg on the PE.

### Cell-matrix detachment during PE elongation

**Fig. 4.**
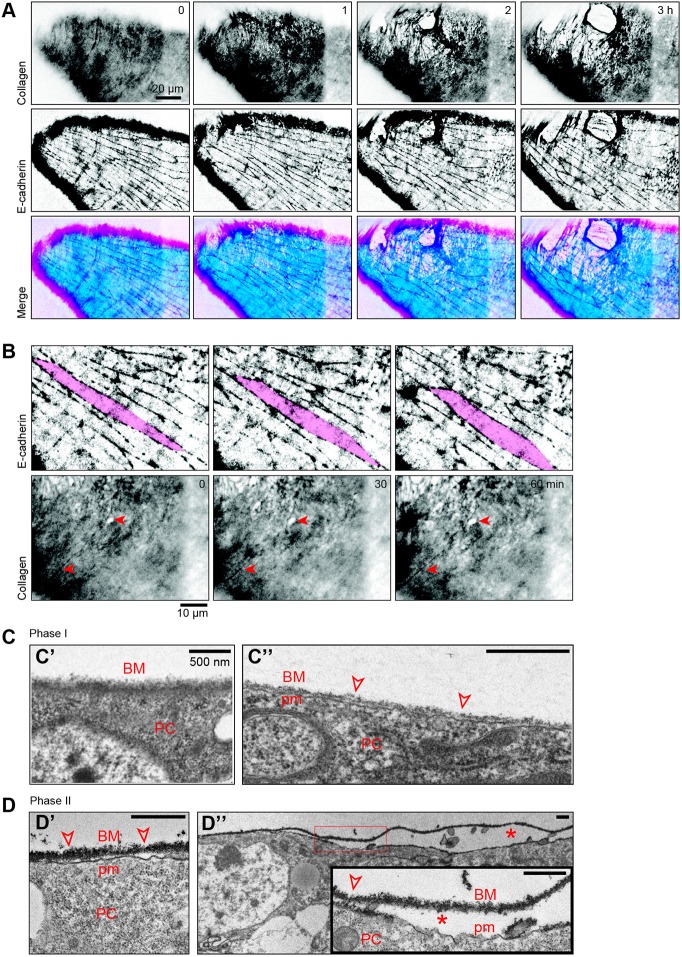
**Physical uncoupling of the ECM from the cell monolayer during PE stretching.** (A) Time-lapse confocal images of the peripodial epithelium of a leg disc (dorsal view) expressing GFP-tagged collagen (*vkg-GFP*) and GFP-tagged E-cadherin (*E-Cad-KI[GFP]*) during late phase I. Top row: single *z*-slice in the plane of the matrix. Middle row: single *z*-slice in the plane of the cell-cell junctions. Bottom row: merged images (cyan, matrix plane; magenta, junction plane). Both reporters are GFP-coupled proteins and color indicates only the *z*-slice. *n*=22/22 leg discs [16/16 matrix and myosin markers (vkg-GFP and *sqh-TagRFPt[9B]*), 6/6 matrix and junction markers (vkg-GFP and E-cad-KI-GFP)] (see also Movie 3). (B) Time-lapse confocal images of the peripodial epithelium of a leg disc (dorsal view) expressing GFP-tagged collagen (*vkg-GFP*) and GFP-tagged E-cadherin (*E-Cad-KI[GFP]*) during late phase I. Top row: single *z*-slice in the plane of the apical junctions (z=−1.5 µm). Bottom row: single *z*-slice in the plane of the matrix (z=0 µm). The cell monolayer follows PE stretching towards the distal region (see magenta cell). *n*=9/9 leg discs (see Movie 4 for higher resolution). Fixed points on the matrix (red arrowheads) show that the matrix is globally static while the cell monolayer slides distal-wards. (C,D) Transmission electron micrographs of the peripodial epithelium from the leg discs of pupae (*w1118*) dissected during phases I (1-2 h APF, *n*=5 legs) and II (3-4 h APF, *n*=6 legs). During phase I (C), cells are in close contact with the matrix (C′) with only subtle cell-matrix detachment (under 50 nm apart, C″, arrowheads). During phase II (D), the matrix bears multiple sites of detachment (over 50 nm apart, D′, arrowheads) and gaps are found between the cell monolayer and the matrix (over 200 nm apart, D″, asterisks). Gaps were observed in 13/62 non-overlapping frames in phase II compared with 0/85 in phase I. Inset corresponds to a higher magnification of the gap framed in red. BM, extracellular matrix; PC, peripodial cell layer; pm, plasma membrane. Scale bars: 500 nm.

The loss of integrity of the PE basement membrane is visible through large matrix holes beneath the cell monolayer. To verify that the cell monolayer remained cohesive despite the holes in the matrix, we imaged fluorescent markers of the cell monolayer and the matrix simultaneously. During hole formation in the matrix, cells maintained their cohesion and appeared not to be affected by the major remodeling beneath them (Fig. 4A and Movie 3, *n*=22/22). Moreover, by following collagen and the intercellular junctions simultaneously, we found that the motion of the cell monolayer and of the matrix layer appeared independent from each other, even in regions where ECM is not yet deteriorated (Fig. 4B and Movie 4, *n*=9/9). This suggested that the cell monolayer and the ECM were physically uncoupled. Consistent with this hypothesis, the level of Talin, a component of focal adhesion, is extremely low in the PE prior to epithelium rupture compared with the level observed in the disc proper and seems to be further reduced at the time of rupture (Fig. S4).

To gain further evidence for this uncoupling, we imaged the basal region of the PE using transmission electronic microscopy. Leg discs were extracted from living prepupae at phase I and II (see Materials and Methods) so that the observations reflect the state of the PE *in vivo*. During phase I, the ECM and the cell monolayer adhered together as expected for regular epithelia (Fig. 4C). In contrast, during phase II, the basal cell region displayed gaps between the cell monolayer and the ECM (Fig. 4D), showing cell-matrix detachment. This did not lead to cell death in the PE (Fig. S5), suggesting that viability of PE cells did not rely on adhesion to the matrix at this stage of leg disc development. Together, these data demonstrate that the PE cell monolayer and its basement membrane uncouple during leg elongation.

### PE opening and retraction are independent of the ECM

Whereas the matrix deteriorates at the distal region, the PE systematically opens at the dorsal tip. As matrix metalloproteinase (MMP)-dependent ECM degradation is involved in wing disc eversion (Srivastava et al., 2007), one could expect that local ECM degradation in the PE could cause a loss of epithelial integrity. However, as the ECM and cell monolayer are uncoupled at the time of PE opening, we decided to test whether PE opening is regulated independently of matrix degradation.

**Fig. 5.**
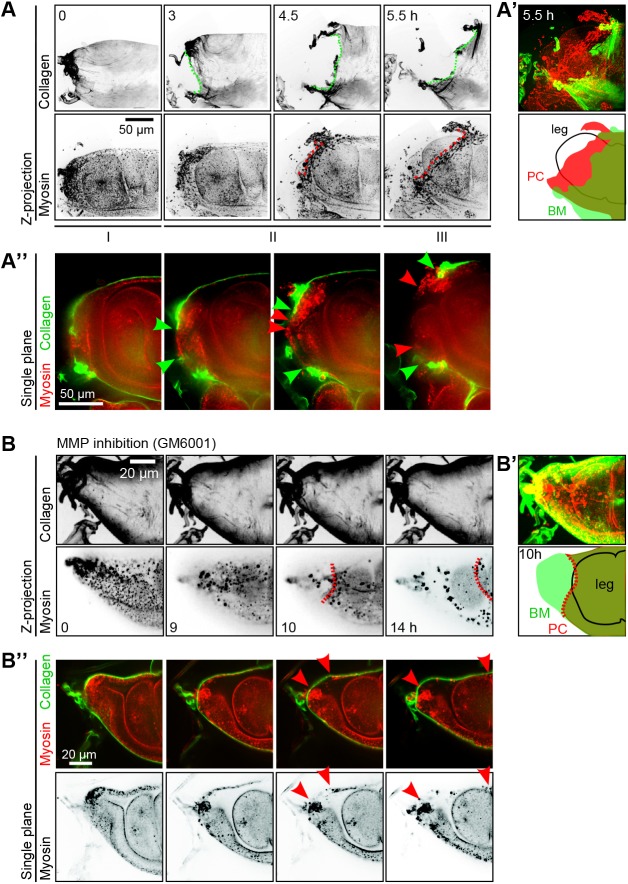
**The PE cell monolayer opens independently of the ECM layer.** (A-A″) Time-lapse confocal images (*z*-projections in A,A′ and *z*-section in A″) of the proximal region of a leg disc expressing fluorescent collagen (*vkg-GFP*) and myosin (*sqh-Tag-RFPt [9B]*) showing that the basement membrane opens before the peripodial epithelium (green dotted line, ECM opening; red dotted line, epithelial opening). Phases I, II and III are indicated beneath the stills. *n*=39 leg discs [collagen (*vkg-GFP*), 34; perlecan (*trol-GFP*), 5] (see also Movie 5). (A′) Top: merged images of collagen (green) and myosin (red) at t=5.5 h showing that the cell layer retraction front is delayed with respect to the ECM layer. Bottom: schemes of the basement membrane (BM, green area), the peripodial cell layer (PC, red area) and the leg (black outline) at this time point. (A″) Single planes of the same time-lapse experiment showing the opening of the ECM layer (green arrowheads) before the opening of the PE monolayer (red arrowheads). (B-B″) Time-lapse confocal images (*z*-projections in B,B′ and *z*-section in B″) of an L3 leg disc (proximal region) expressing fluorescent collagen (*vkg-GFP*) and myosin (*sqh-TagRFPt[9B]*), cultured in DMSO (0.5%) with an MMP inhibitor (GM6001, 50 µM) (*n*=11/11). MMP inhibition preserves ECM integrity at the dorsal tip. However, the peripodial cell monolayer opens and retracts (dotted red outline) at the dorsal tip, despite MMP inhibition (see also Movie 6). Control L3 leg disc cultured in DMSO is shown in Fig. S6. (B′) Merged images (top) of collagen (green) and myosin (red) at *t*=10 h, and corresponding schemes of the basement membrane (BM, green area), the peripodial cell monolayer (PC, red area) and the leg disc proper (black outline). The retraction front of the cell monolayer is outlined in dotted white or red. (B″) Single planes of the same time-lapse experiment showing the opening of the PE monolayer (red arrowheads) beneath the intact ECM layer.

We first characterized the behavior of the PE matrix at the opening site by imaging ECM dynamics in this particular region. A hole in the ECM layer appears during phase I at the dorsal tip of the PE (Fig. 5A and Movie 5). Strikingly, this hole does not disrupt the cell monolayer, which always opens later (Fig. 5A and Movie 5). This suggests that the matrix and the cell monolayer behave independently at the time of opening. To inquire into this independent behavior, we sought to prevent the formation of a hole in the ECM.

For this purpose, we cultured the tissues in the presence of the broad-spectrum MMP inhibitor GM6001 and followed the dynamics of the PE. When tissues were subjected to MMP inhibition in phase I, at the start of elongation, the matrix was degraded normally, the PE retracted progressively and the leg everted in the same way as control legs (data not shown). Nevertheless, we checked whether the matrix degradation process was not anterior to elongation by culturing tissues at the end of the third larval stage, i.e. before phase I. In the presence of ecdysone, the third-instar larval leg elongates as well as its PE, albeit less than a prepupal leg tissue (control DMSO, see Fig. S6). In the presence of the MMP inhibitor, ECM degradation at the dorsal tip was prevented throughout tissue elongation (Fig. 5B). This suggests that ECM degradation results from local MMP activity before the start of phase I.

Interestingly, although the ECM in the PE was intact, the PE cell monolayer opened at the same location as in control conditions (Fig. 5B and Movie 6, *n*=11/11). This demonstrates that, in this tissue, the opening of the PE monolayer is independent of matrix degradation, and constitutes additional evidence that the monolayer behaves independently from the matrix.

**Fig. 6.**
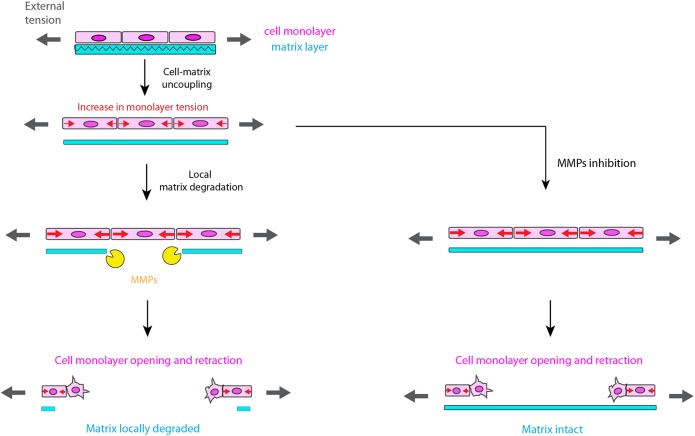
**Physical and functional uncoupling of the ECM from the cell monolayer in an epithelium under tension.** First, the epithelium is under tension (black arrows), which is mostly borne by the ECM (cyan). Then the cells and the matrix become uncoupled and tension increases in the monolayer (red arrows). Independently of matrix degradation (presence or absence of MMPs), the monolayer opens, possibly through local EMT, and retracts through myosin-dependent contraction.

Overall, our results show that the mechanics and the organization of the PE change drastically during leg elongation. The PE cell monolayer and the ECM appear uncoupled on two levels. On the one hand, the ECM detaches from the cell monolayer, which displays an increase in tension and contractility, and then retracts (Fig. 6). On the other hand, the opening of the monolayer does not require ECM degradation, which suggests that PE cell behavior is independent of the ECM.

## DISCUSSION

In this study, we have analyzed the mechanical properties dynamics of an epithelium naturally put under tension during development. We focused on the peripodial epithelium of the *Drosophila* imaginal leg disc, and found that during PE elongation, tension is at first mainly borne by the basement membrane, then shared with the cell monolayer at the end of the elongation phase. Strikingly, this change in the mechanical state of the monolayer is concomitant with a loss of cell-matrix interaction, and both layers follow independent paths from this stage. Indeed, after reaching maximal length, the monolayer opens independently of matrix degradation and retracts autonomously in a myosin-dependent manner. These results support a model in which cell-matrix disengagement may favor an active retraction of the cell monolayer. Thus, cell-matrix uncoupling could act as a developmental timer, and hence constitute an alternative to classical hormonal signals for the control of stereotyped organ morphogenesis.

In the *Drosophila* wing disc, the PE opening process has been associated with a local epithelial-mesenchymal transition (EMT)-like cell behavior, production of MMP and reduction of cell-cell adhesion (Pastor-Pareja et al., 2004; Srivastava et al., 2007). In this perspective, PE cells located at the dorsal tip would launch ECM proteolysis, undergo EMT and reduce their adhesion to break free from their neighbors and migrate to the larval epidermis. However, the respective contribution of the ECM and the cell monolayer during leg evagination was never considered. Our work brings new insights into the mechanical contribution of each component to PE dynamics. Although it was tempting to speculate that EMT could be a direct consequence of ECM local degradation, we observe, surprisingly, that ECM proteolysis at the dorsal tip is not required for the opening and retraction of the PE, although it is undoubtedly necessary, so that the leg may evert after PE retraction. Our results further point at a role of MMPs before the beginning of leg elongation, suggesting that local production of MMPs at the dorsal tip could prepare the ECM for a subsequent opening that may result from the mechanical force exerted by the elongating leg. Finally, our work reveals the respective contribution of the cell monolayer and the ECM in tissue retraction with a 1st phase of collaboration, the ECM supporting the tension, and a 2nd phase of uncoupling and independent removal. We propose that the shift between these two steps may force PE cells to withstand higher amounts of tension by increasing their acto-myosin contractile machinery.

These results also reveal that epithelia can survive *in vivo* in the absence of cell-matrix adhesion. Indeed, while ECM is progressively degraded and the PE monolayer uncoupled from the basement membrane, the PE preserves its structure, and its cells remain cohesive and survive. Although the lack of cell-matrix adhesion is known to induce cell death by apoptosis in single adherent cells (Chen et al., 1997), recent work has shown that a monolayer epithelium can be maintained *in vitro* without matrix, at least under tension (Harris et al., 2012; Wyatt et al., 2015). Our observations indicate for the first time that this is also true *in vivo*. Furthermore, they suggest that, in a monolayer epithelium, the maintenance of intercellular adhesion might compensate for basal adhesion loss. Indeed, we were able to detect apoptosis essentially at the free edge after PE opening, where cells have not only lost adhesion to the ECM but also to part of their cell-cell adhesion complexes. This supports the notion of a survival signal from intercellular adhesion in an epithelium under tension, through a combination of signaling from adherens junction-linked proteins and mechano-transduction (Discher et al., 2009; Janmey et al., 2009). Further studies are needed to determine whether this feature is a general property of epithelia.

Most studies of the dynamics of epithelia to date have focused either on the epithelial cells or on the matrix. However, both are essential constituents of epithelia that differ notably in their behavior and mechanical response, and that confer specific physical properties to tissues. Each cell can modify its shape and rigidity actively through the reorganization of cytoskeletal components and the generation of intracellular forces by molecular motors such as myosin. As for the matrix, because it does not contain any motor proteins, it is considered to be a passive element characterized by its composition and architecture. However, the present study argues that the dynamics of ECM and, in particular, its interplay with the cells can have a major impact on morphogenesis. Here, we benefitted from the particular geometry of the PE (a flat epithelium supported by a basement membrane forming the outer layer of the leg disc and thus directly accessible) and from the fact that *Drosophila* leg tissues can accomplish their morphogenesis in culture. We could decipher the relative contribution of matrix and epithelial cells to tissue mechanics during a long-scale morphogenetic process. Combining live imaging with biophysical approaches, we show that the PE cell monolayer and the ECM behave independently in terms of tension, opening and withdrawal, and propose that they respond to different signals, either biochemical or mechanical. Although the layers constituting an epithelium are generally viewed as interdependent, our work reveals that, surprisingly, they can become uncoupled in a developmental context. This physical and functional uncoupling of the ECM and the cell monolayer was unexpected, and highlights the necessity to revisit the classical vision of epithelia.

## MATERIALS AND METHODS

### Fly stocks

sqh-eGFP[29B] and sqh-TagRFPt[9B] (this work) are knock-in designed and generated by homologous recombination by InDroso functional genomics. The respective tags were inserted in the C-terminal just before the stop codon and the resulting flies were validated by sequencing.

ECadherin-GFP is a knock-in line (Huang et al., 2009). sqh::sqh-GFP on the 2nd chromosome (Royou et al., 2002) and UAS::GC3ai (Schott et al., 2017) have been previously described. vkg-GFP[G0454] and Trol-GFP[G00022] are Flytrap lines (Morin et al., 2001). Rhea-mCherry[MI00296], C855a-Gal4 [6990] and uas::zip-RNAi[7819] were obtained respectively from BDSC and VDRC. w[1118] flies were used for transmission electron microscopy experiments.

### Sample preparation

Leg discs were dissected from L3 larvae, white pupae (WP, 0-1 h after puparium formation or APF) or 2-3 h APF pupae in Schneider's insect medium (Sigma-Aldrich) supplemented with 15% fetal calf serum and 0.5% penicillin-streptomycin, as well as 2 µg/ml 20-hydroxyecdysone (Sigma-Aldrich, H5142). Leg discs were transferred on a glass slide in 13.5 µl of this medium confined in a 120 µm deep double-sided adhesive spacer (Secure-Seal from Sigma-Aldrich) and a glass coverslip was then placed on top of the spacer. Halocarbon oil was added on the sides of the spacer to prevent dehydration. Dissection tools were cleaned with ethanol before dissection. For MMP inhibition experiments (Fig. 5B, Movie 6), leg discs were mounted in medium supplemented with 50 µM GM6001 and 0.5% DMSO (Abcam, ab120845) or with 0.5% DMSO only (control condition). For myosin inhibition experiments (Fig. S2), C855a-Gal4 and uas::zip-RNAi flies were crossed at 25°C and larvae were grown at 18°C to limit Gal4 activity and avoid disrupting tissue integrity.

### Live microscopy and imaging

Leg disc development was imaged using an inverted spinning disk confocal microscope (CSU-X1, Yokogawa, coupled to a Leica or Zeiss microscope) mounted with 20×/0.8 multi-immersion 40×/1.2 oil or 20×/0.8 air objectives and equipped with 488 nm and 561 nm LEDs and a piezo stage. Images were acquired over time with an EMCCD camera (Hamamatsu) controlled by the Metamorph or Zen software, at a rate of one *z*-stack every 5 to 15 min. Images were processed with the ImageJ software for registration (StackReg plug-in from Thévenaz and co-workers, EPFL, Switzerland), bleaching correction by histogram matching (Bleach Correction plug-in from Miura, EMBL, Germany), stitching (Pairwise Stitching plug-in Preibisch et al., 2009, background correction and smoothing. The length of the PE and that of the leg (Fig. 1C and Fig. S1) were measured on image *z*-stacks, from the dorsal tip to the distal pole (PE) and from the femur to the distal pole (leg), respectively, as indicated in Fig. 1B. The occurrence time of a particular event (Fig. 1D) was defined as the first timepoint when the event was visible.

### Laser dissection

Leg discs were dissected from pupae at 0-1 h APF and 2-3 h APF, which corresponded to phases I and II of leg disc eversion, and mounted between a glass slide and a precision glass coverslip (Marienfeld). Laser dissection experiments were performed with a pulsed DPSS laser (532 nm, pulse length 1.5 ns, repetition rate up to 1 kHz, 3.5 µJ/pulse) steered by a galvanometer-based laser scanning device (DPSS-532 and UGA-42, from Rapp OptoElectronic). The laser beam was focused through an oil-immersion lens of high numerical aperture (Plan-Apochromat 63×/1.4 Imm Oil or LD LCI Plan-Apochromat 63×/1.2 multi-Imm, from Zeiss). Photo-disruption was produced in the focal plane by illuminating between 60% and 100% laser power. To target intracellular structures, illumination duration was set between 40 ms and 2 s on a small spot or a small line focused on an actin-myosin filament or cell-cell junction at 2× zoom. For the ECM layer, 20-40 µm lines were illuminated for 50-100 ms at zoom 1× or 2.8 s at zoom 2×. Images were acquired every 200 ms to 400 ms during the experiment using a confocal laser scanning microscope (LSM-880, Zeiss) equipped with a 488 nm Argon laser and a GaAsP photomultiplier. We checked that cutting cytoskeletal filaments did not disrupt the ECM layer (not shown).

Laser dissection experiments were analyzed assuming a standard Kelvin-Voigt viscoelastic model of the actin-myosin fiber, i.e. with a single fiber stiffness *k* and a single viscous drag coefficient (Kumar et al., 2006). Following this model, tension *F* in the fiber relates to the maximum recoil distance after dissection *L* as *F=k L*. Maximum recoil distances were measured on kymographs of the dissected filament using ImageJ in phase I and phase II (*L_I_* and *L_II_*). From the accumulation of myosin II in the dorsal part of the PE (Fig. 1E), we postulate an increase in fiber stiffness from phase I to phase II, i.e. *k_I_*<*k_II_*. Recoil measurements yield *L_I_*<*L_II_* (Fig. 2C), from which we derive *F_I_=k_I_ L_I_*<*k_II_ L_II_=F_II_*. Therefore, we may conclude that tension in the PE is higher in phase II compared to phase I.

### Transmission electron microscopy

Prepupae (1-2 h APF) and pupae (3-4 h APF) were opened and fixed for 2 h at room temperature with 2.5% glutaraldehyde and 2% paraformaldehyde in 0.08 M cacodylate buffer containing 0.05% CaCl_2_ (pH 7.2, EMS). Leg discs were then dissected and post-fixed at room temperature with 1% OsO_4_ in the same buffer. Samples were treated for 1 h with 1% aqueous uranyl acetate and were then dehydrated in a graded ethanol series followed by 100% acetone. Samples were flat embedded in EMBed-812 resin (EMS). After 48 h of polymerization at 60°C, ultrathin sections (80 nm) were mounted on single-slot Formvar-coated copper grids. Sections were stained with Uranyless (Delta Microscopies) and 3% Reynolds lead citrate (Chromalys). Grids were examined with a TEM (Jeol JEM-1400) at 80 kV. Images were acquired all along the PE in each section using a digital camera (Gatan Orius). Magnifications are indicated in the legend (Fig. 4C,D).

### Statistics

The statistical significance of the difference between occurrence times of particular events (Fig. 1D) was assessed using the one-sided Wilcoxon signed-rank test. Absolute time measurements were paired by leg disc. The null hypothesis was that the time difference values between PE opening and PE contraction were samples from a symmetric distribution centered below zero. The significance of the difference between recoil distances after laser dissection (Fig. 2C and Fig. S3C) was assessed using the Mann–Whitney test. The null hypothesis was that recoil distance during phase I was greater than during phase II. *P*-values were computed with the R software and significance is indicated as follows: *P<0.05, **P<0.01, ***P<0.001.

## Supplementary Material

Supplementary information
