# Supplementary material for: Physical and functional cell-matrix uncoupling in a developing tissue under tension
Source: Development. 2019 Jun 3;146(11):dev172577. doi: 10.1242/dev.172577 (PMC6589077; doi:10.1242/dev.172577)
Supplement: Supplementary information [file develop-146-172577-s1.pdf]

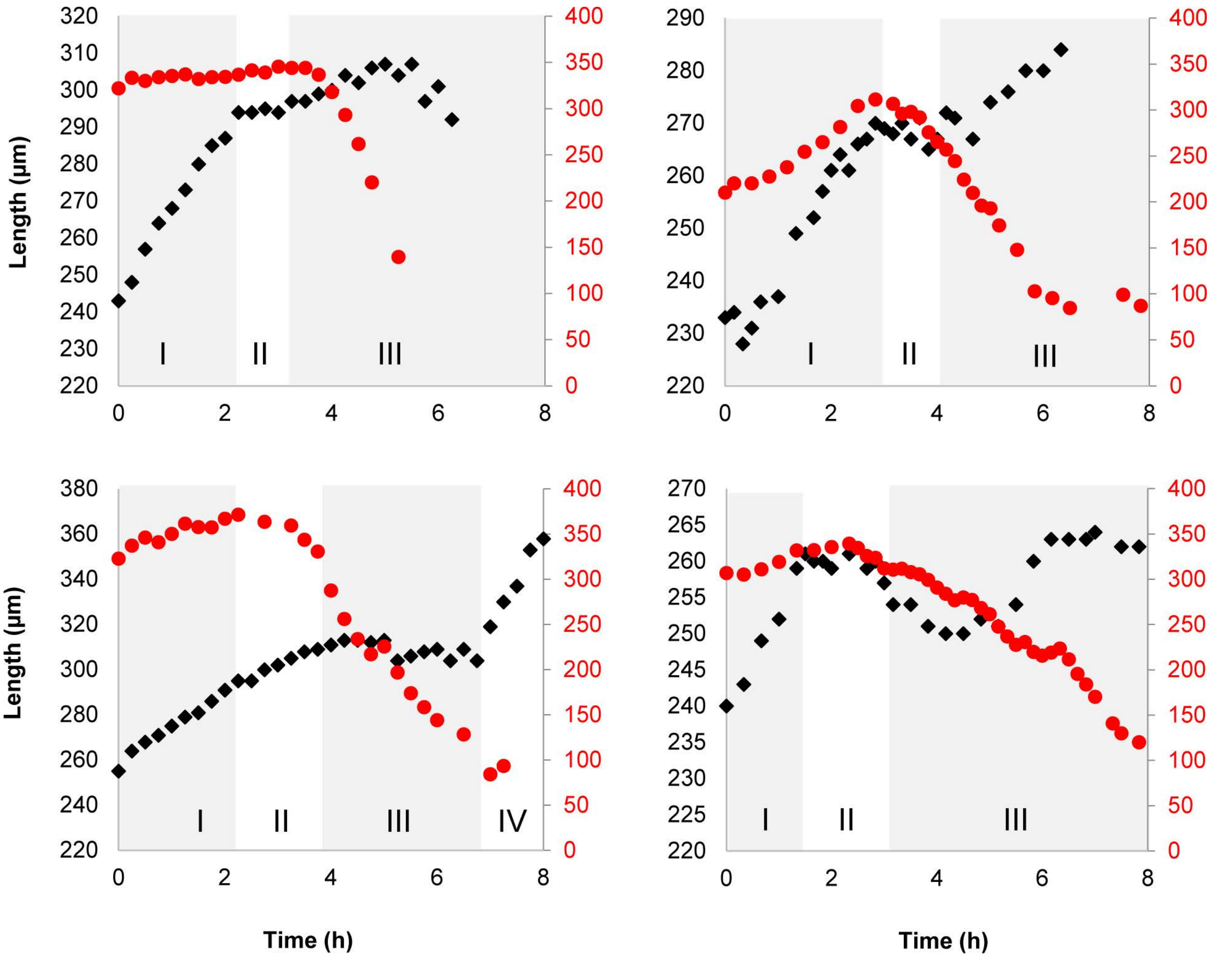

Figure S1: Related to Figure 1. Envelope dynamics during leg elongation.

Dynamics of leg elongation and envelope stretching for 4 leg discs. Length of the leg (black) and of the envelope (red) over time, measured as indicated on **Fig1b**. Phases I-IV are described in the text.

Development • Supplementary information

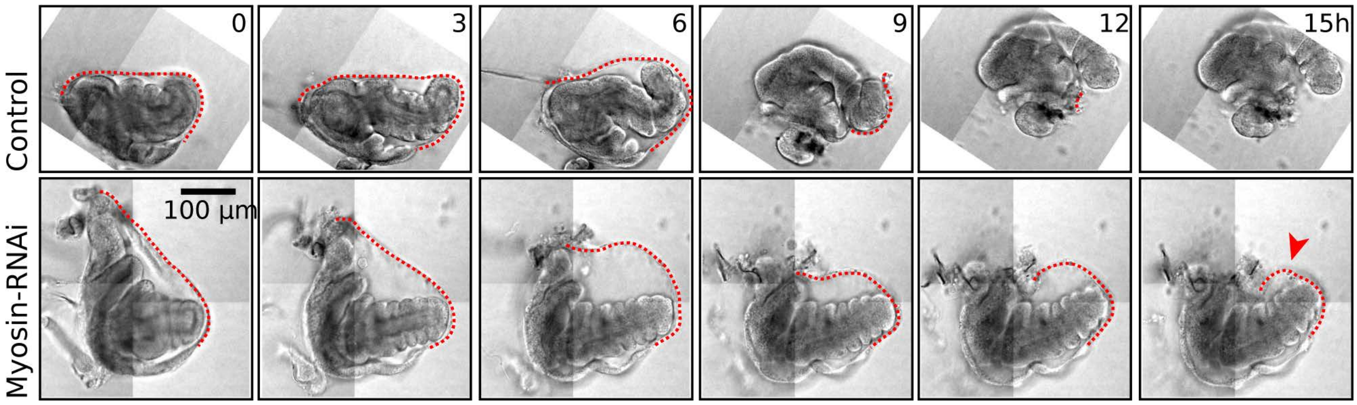

Figure S2: Myosin activity is required for PE retraction.

Time-lapse bright-field images of a leg disc expressing myosin-RNAi in the PE (*c855a-Gal4 x uas::zip-RNAi*, bottom row) compared to a control leg disc (*uas::zip-RNAi*, top row). Myosin depletion prevents retraction of the PE. The PE is outlined in dashed red over time. Retraction was impaired for7/7 leg discs.

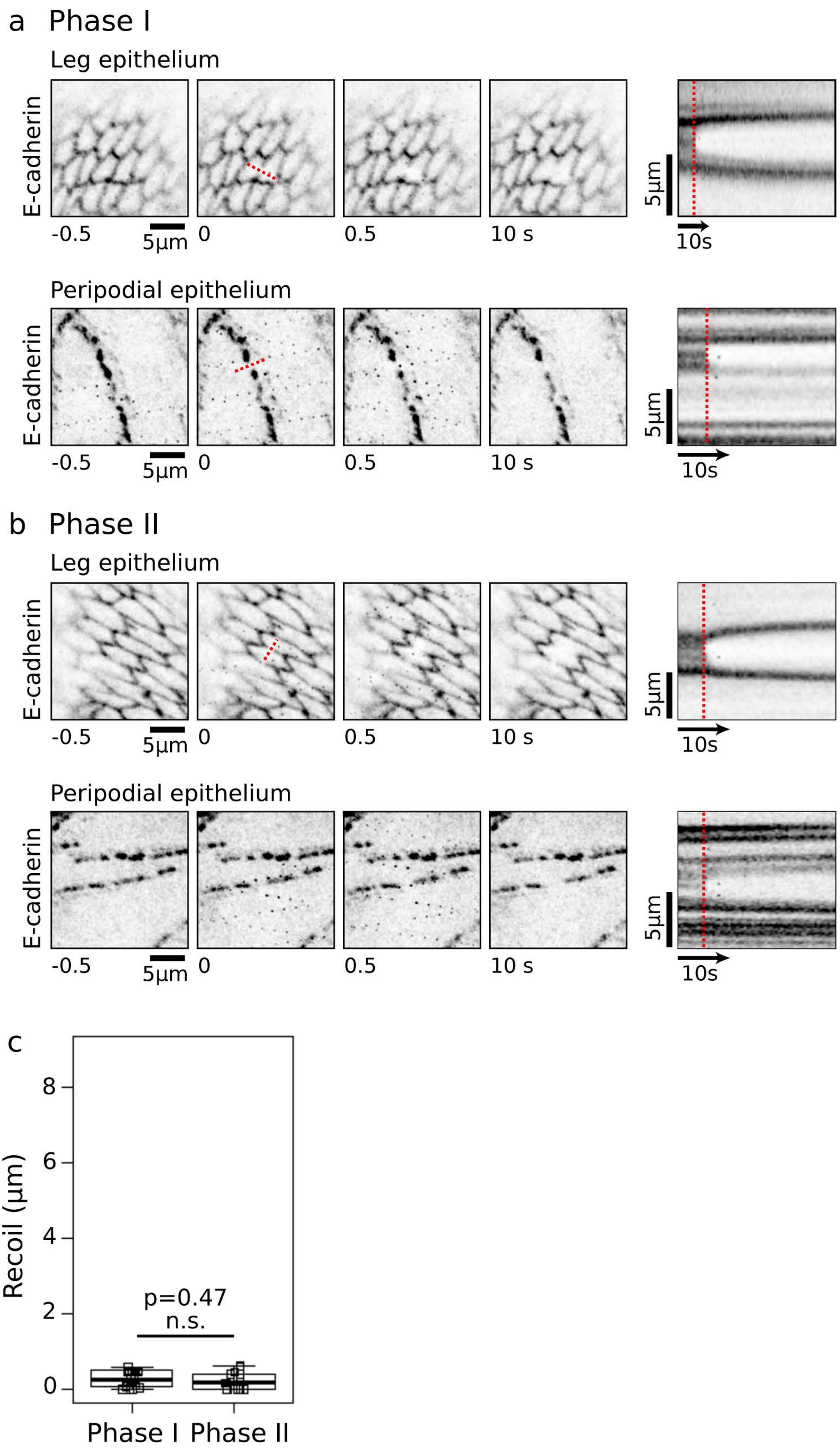
a Phase I
Leg epithelium
E-cadherin
-0.5
5μm
0
0.5
10 s
5μm
10s
Peripodial epithelium
E-cadherin
-0.5
5μm
0
0.5
10 s
5μm
10s
b Phase II
Leg epithelium
E-cadherin
-0.5
5μm
0
0.5
10 s
5μm
10s
Peripodial epithelium
E-cadherin
-0.5
5μm
0
0.5
10 s
5μm
10s
c
Recoil (μm)
8
6
4
2
0
p=0.47
n.s.
Phase I
Phase II

Figure S3: Related to Figure 2. Laser dissection of adherens junctions in the PE and the leg epithelium.

a. Laser dissection of a cell-cell junction in the disc proper (top row) and the peripodial epithelium (bottom row) of a leg disc in phase I expressing fluorescent E-cadherin (*E-Cad-KI[GFP]*). Time-lapse confocal images are shown in one single plane. Dotted red line indicates laser illumination. Rightmost panel: kymograph along the junction, showing recoil dynamics (dotted red line denotes the illumination time point). Representative of 7/7 leg discs. Although the experimental conditions are adequate to dissect junctions in the disc proper, peripodial junctions do not recoil. Hence laser dissection of cell-cell junctions is not hampered by the basement membrane of the peripodial epithelium and the lack of peripodial junction recoil indicates low tension or stiffness of the structure.

b. Same experiment as **a**, for a leg disc in phase II. Representative of 5/5 leg discs.

c. Recoil distance for PE cell-cell junctions dissected in phases I (n=17 junctions) and II (n=14) are comparable. Note that the scale of the graph is the same as that of myosin filament recoil after ablation (see Fig2c).

Development • Supplementary information

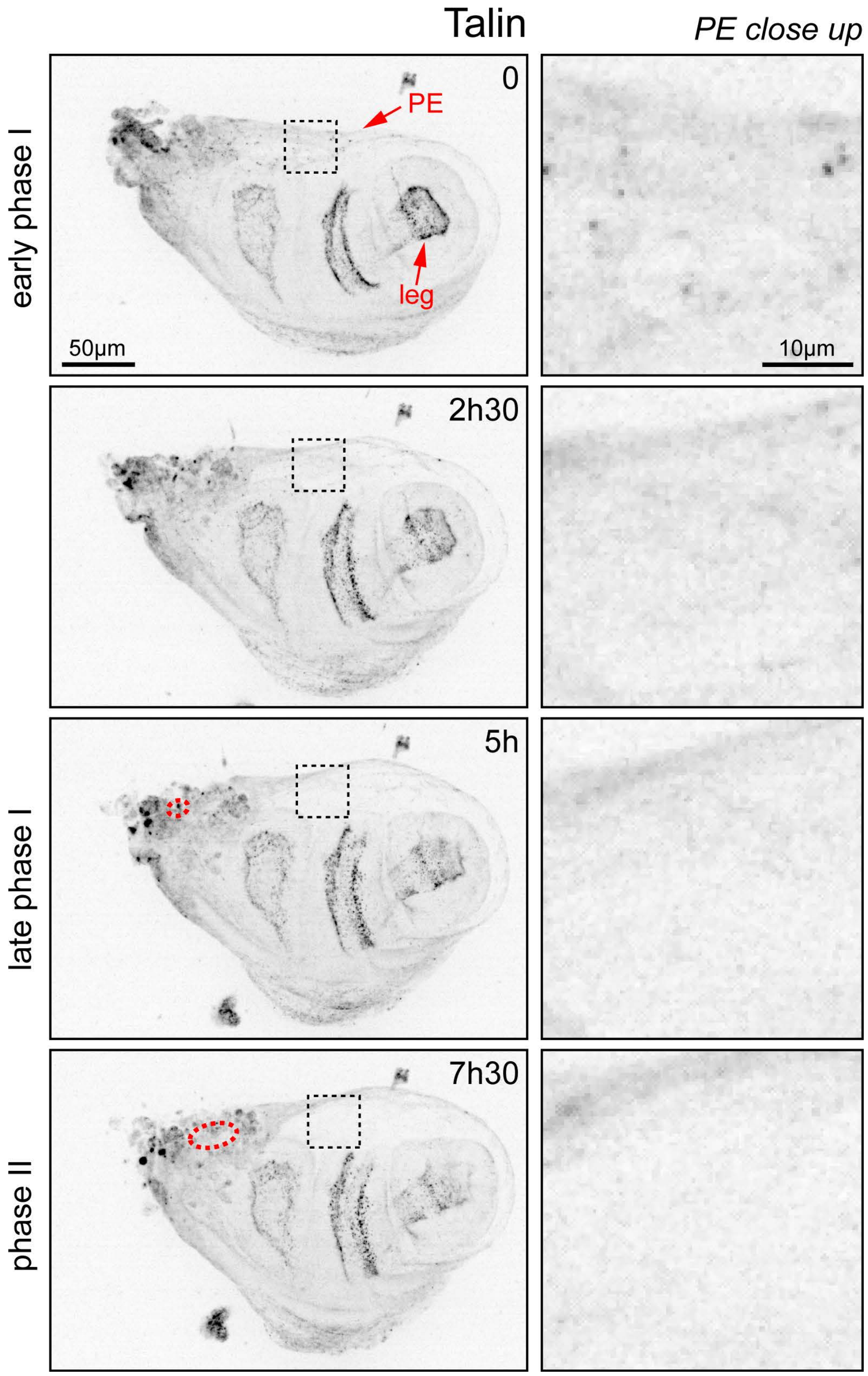

Figure S4: Cell-matrix adhesion is reduced while the PE breaks open.

Time-lapse images (z-projection) of a leg disc expressing a Talin-mCherry fusion protein at the transition between phase I and II. Note the low level of expression of Talin in the PE, which appears to be further reduced at the time of PE opening (close up, right panels), compared to the strong expression detected in the leg. Representative of 5/5 leg discs. PE opening (red dotted circles) is based on the Myosin-GFP channel (not shown).

Development • Supplementary information

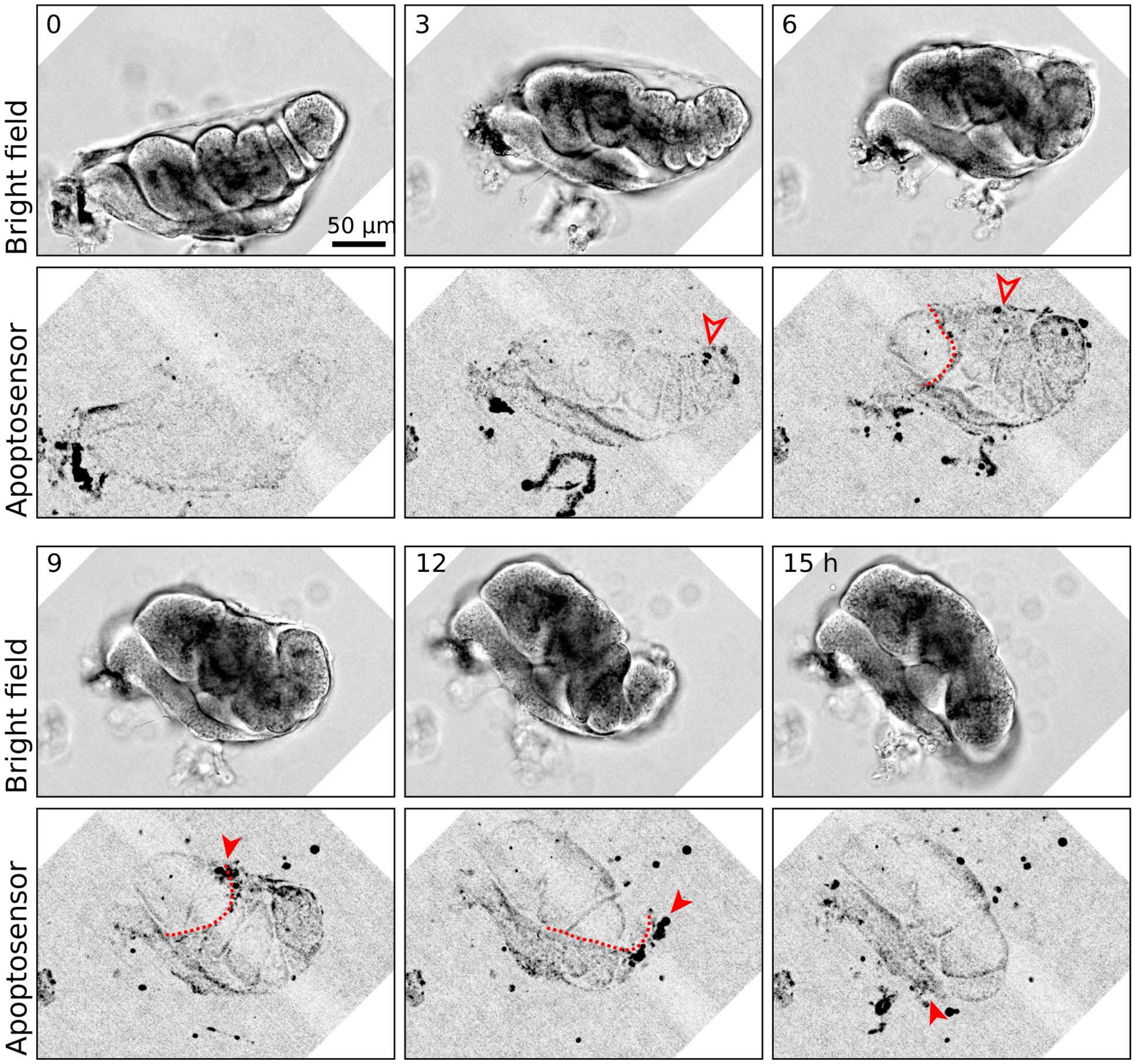

Figure S5: Apoptosis is restricted to the retraction front of the PE.

Time-lapse images of a leg disc expressing a fluorescent apoptosis reporter in the PE (*c855a-Gal4 x uas::GC3Ai*). Top rows: bright field (single plane); bottom rows: apoptosensor (z-projection). Apoptosis is sporadically detected in the PE (open arrowhead) and then mostly at the retraction front (filled arrowheads). The PE retraction front is outlined in dashed red over time. Phenotype observed on 5/5 leg discs.

Development • Supplementary information

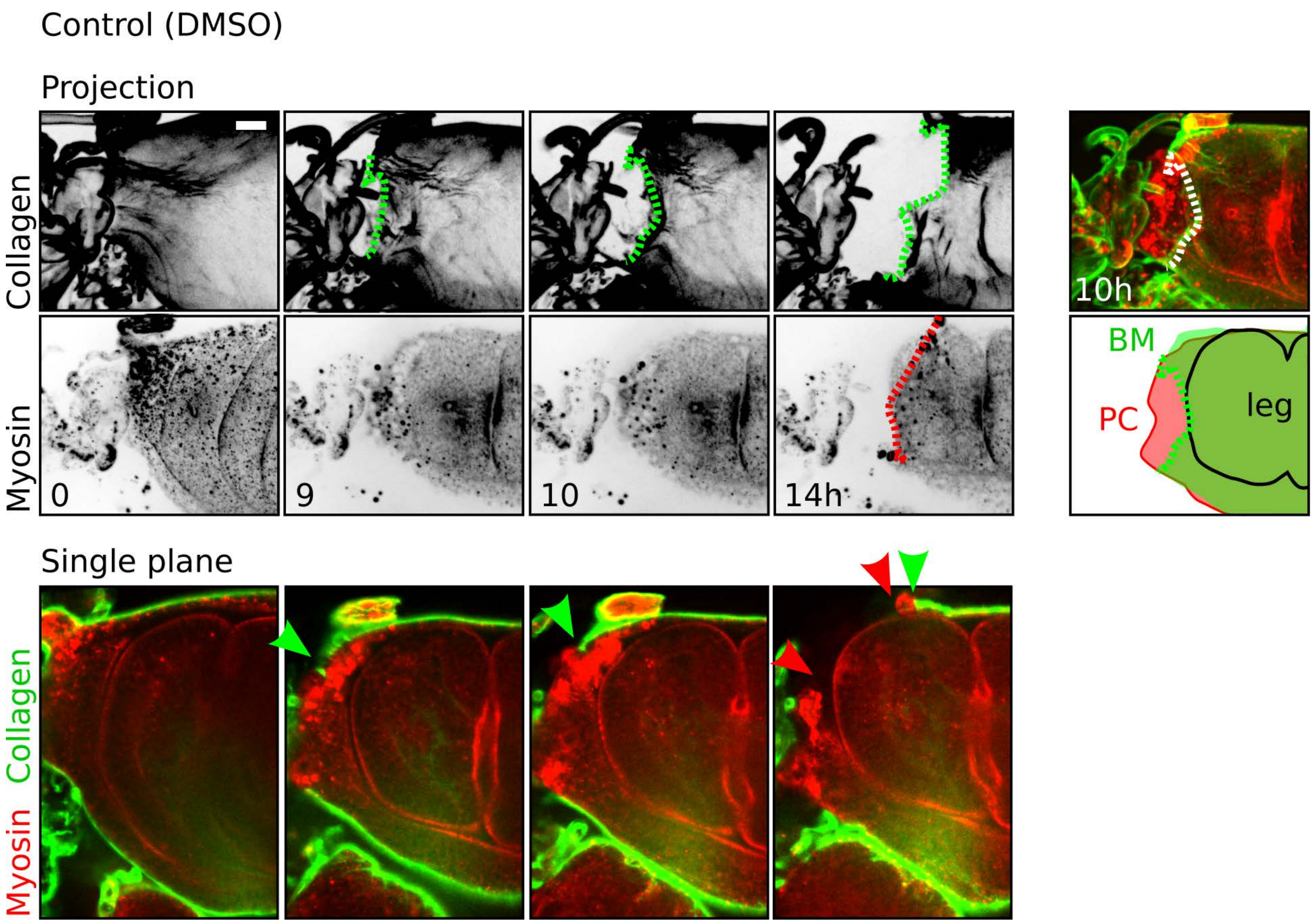

Figure S6: Related to Figure 5. PE and ECM opening in control L3 leg discs .

Time-lapse confocal images (top row: z-projections, bottom row: single plane) of the proximal region of a third instar leg disc expressing fluorescent collagen (*vkg-GFP*) and myosin (*sqh-TagRFPt[9B]*), cultured in DMSO (0.5 %) (n=7/7). Presence of ecdysone in the culture medium induces leg elongation. Similarly to cultured prepupal leg discs, the ECM layer opens at the dorsal tip and retracts (dotted green outline). The peripodial cell monolayer opens and retracts (dotted red outline) at the dorsal tip slightly later. See also **Movie 6**. On the right are shown merged images (z-projections) of collagen (green) and myosin (red) at *t* = 10 h and corresponding schemes of the basement membrane (**BM**, green area), the peripodial cell monolayer (**PC**, red area) and the leg disc proper (black outline). The retraction front of the basement membrane and of the cell monolayer are outlined in dotted green (or white) and red respectively on projections and shown with green and red arrows on single planes of the same time-lapse experiment. The opening of the ECM layer takes place before that of the PE monolayer. Compare to the inhibition of MMPs presented in Fig5b.

Development • Supplementary information

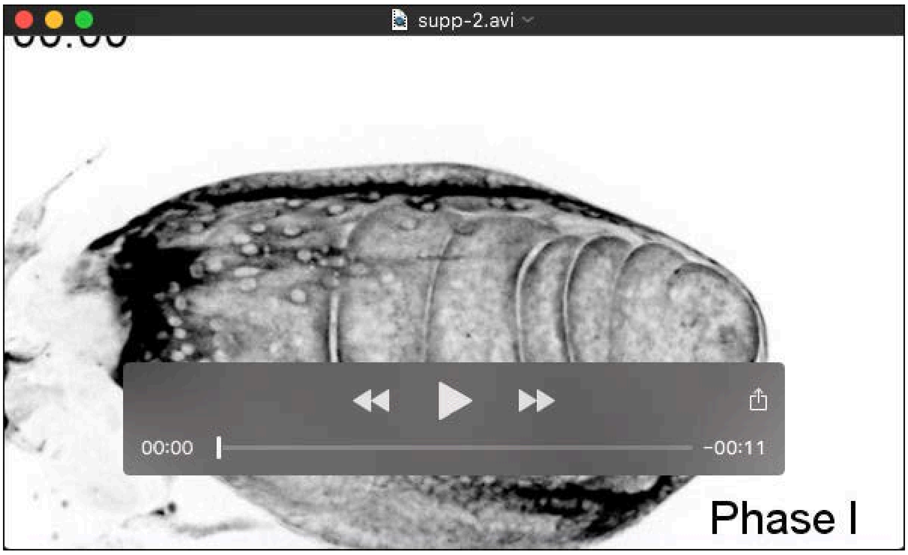

Movie 1. Related to Figure 1b. Peripodial epithelium elongation, tearing and removal during leg elongation.

Leg disc eversion in culture. Time-lapse confocal microscopy images (z-projections) of a leg disc expressing fluorescent myosin. Tissue orientation follows Fig1a (dorsal-ventral from top to bottom, proximal-distal from left to right). Image stacks were acquired every 10 min.

Development • Supplementary information

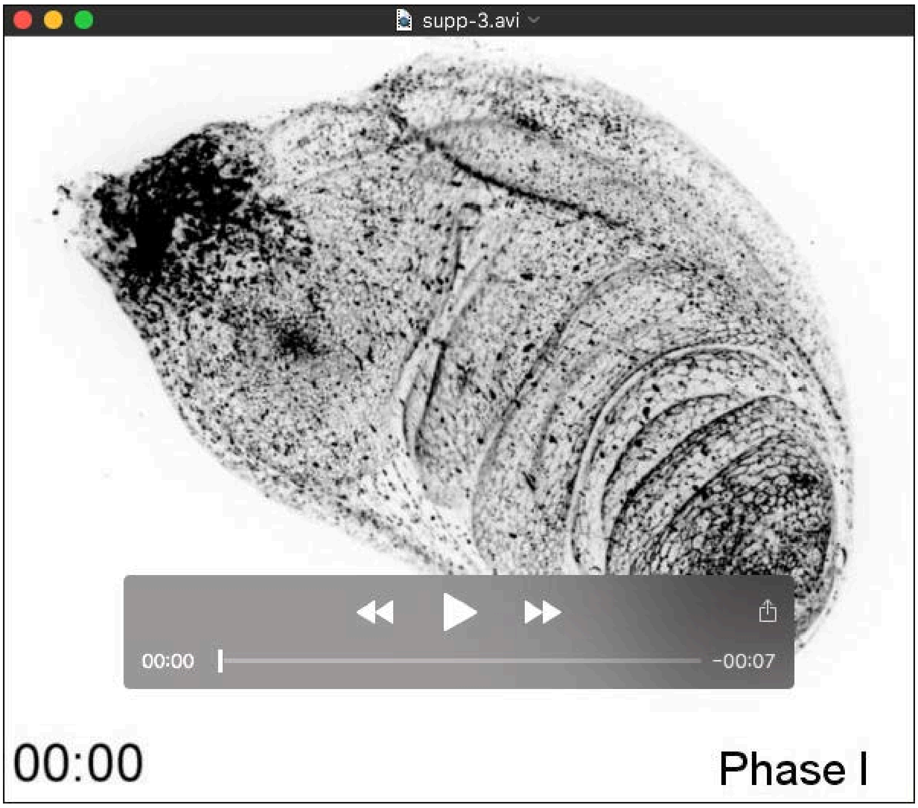

Movie 2. Related to Figure 1e. Myosin in the peripodial epithelium organizes into cable-like structures before PE contraction and opening.

Time-lapse confocal images (z-projection) of the opening site of a leg disc expressing fluorescent myosin (*sqh-TagRFPt[9B]*). Image stacks were acquired every 5 min.

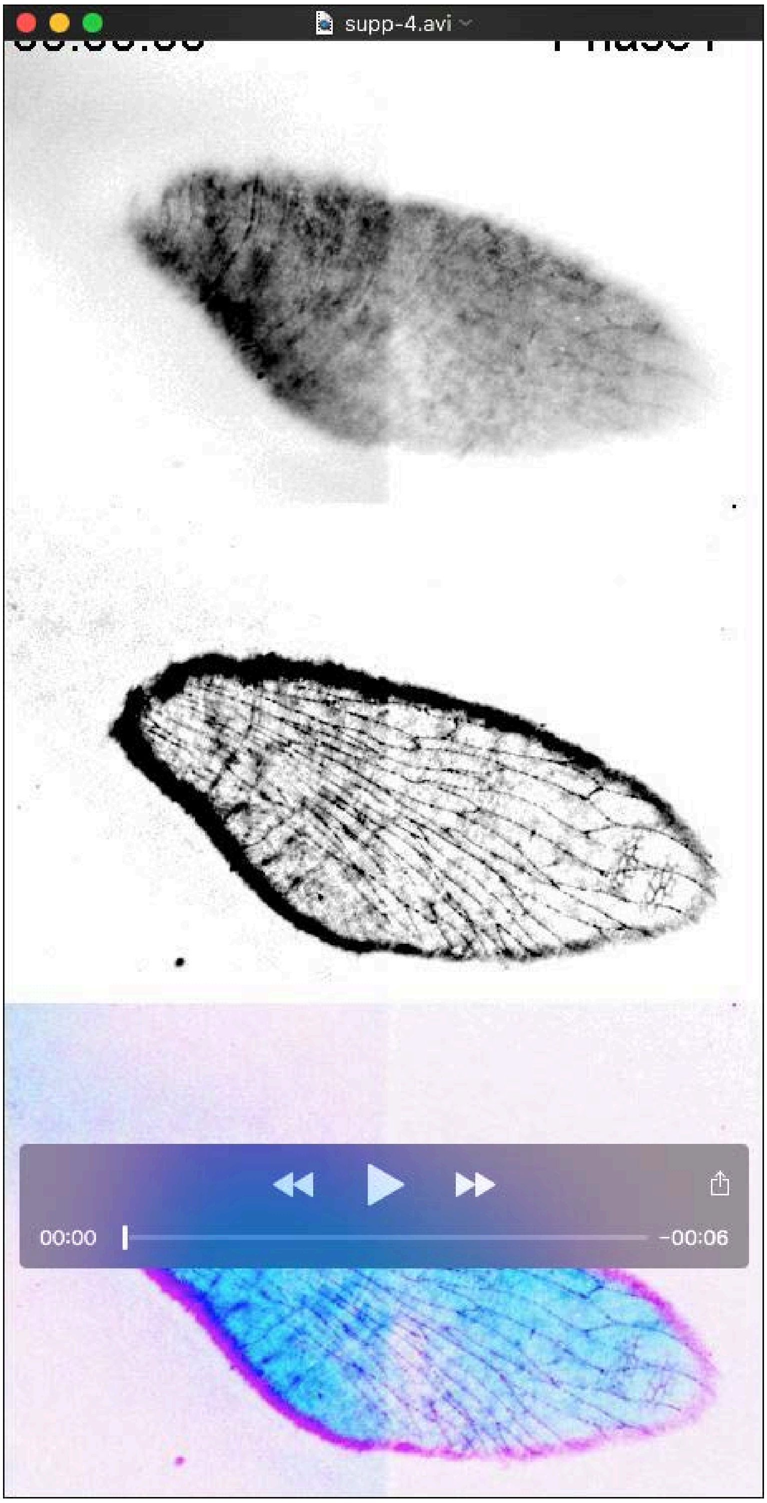

Movie 3. Related to Figure 4a. Peripodial cells remain cohesive despite deterioration of the matrix layer.

Time-lapse confocal images of the peripodial epithelium of a leg disc (dorsal view) expressing GFP-tagged collagen (*vkg-GFP*) and GFP-tagged E-cadherin (*E-Cad-KI[GFP]*).Top: matrix plane; middle: cell-cell junction plane; bottom: merged images (matrix plane: cyan, cell-cell junction plane: magenta). Image stacks were acquired every 3.75 min.

Development • Supplementary information

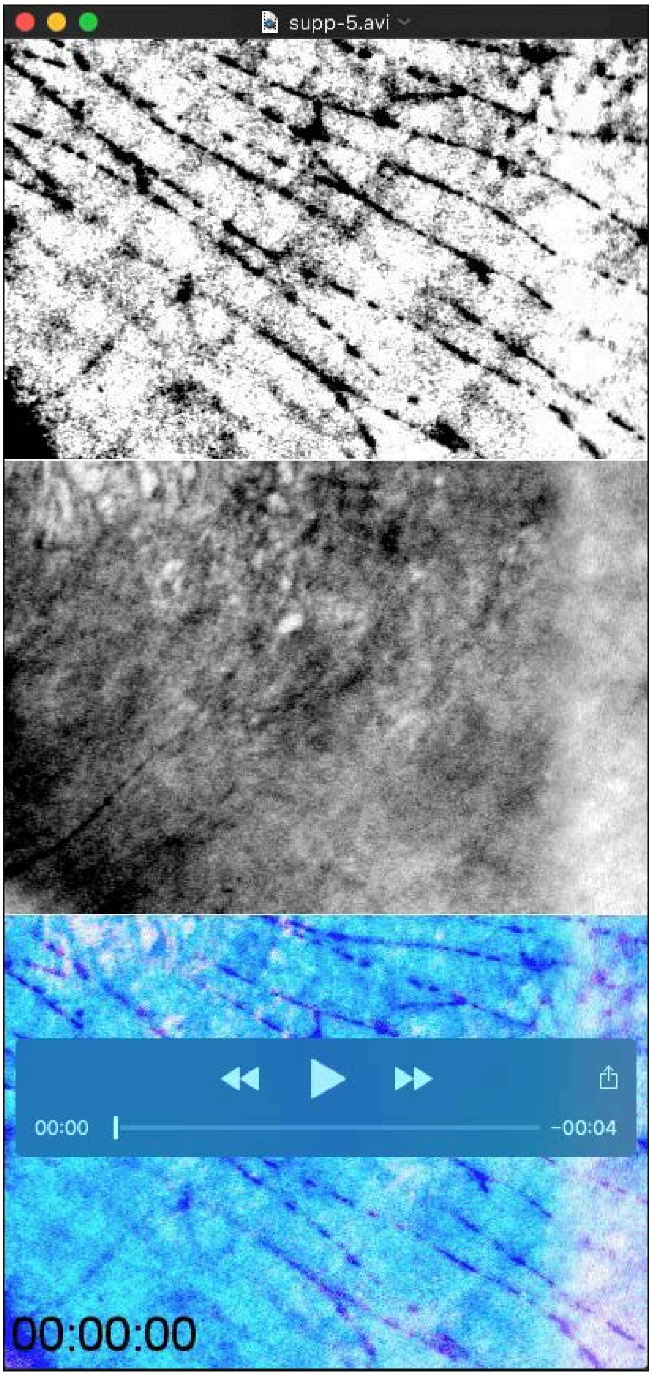

Movie 4. Related to Figure 4b. Relative sliding of the cell monolayer and the matrix.

Time-lapse confocal images of the peripodial epithelium of a leg disc (dorsal view) expressing GFP-tagged collagen (*vkg-GFP*) and GFP-tagged E-cadherin (*E-Cad-KI[GFP]*).Top: cell-cell junction plane; middle: matrix plane; bottom: merged images (collagen: cyan, E-cadherin: magenta). Image stacks were acquired every 3.75 min.

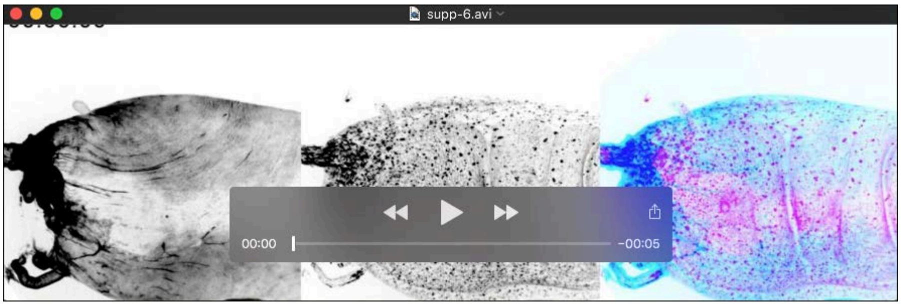

Movie 5. Related to Figure 5a. Uncoupling of the ECM from the cell monolayer during peripodial epithelium opening.

Time-lapse confocal images (z-projections) of a leg disc expressing fluorescent collagen (*vkg-GFP*, left) and myosin (*sqh-TagRFPt[9B]*, center) during phases I-III. Right: merged images (cyan: collagen, magenta: myosin). Image stacks were acquired every 7.5 min.

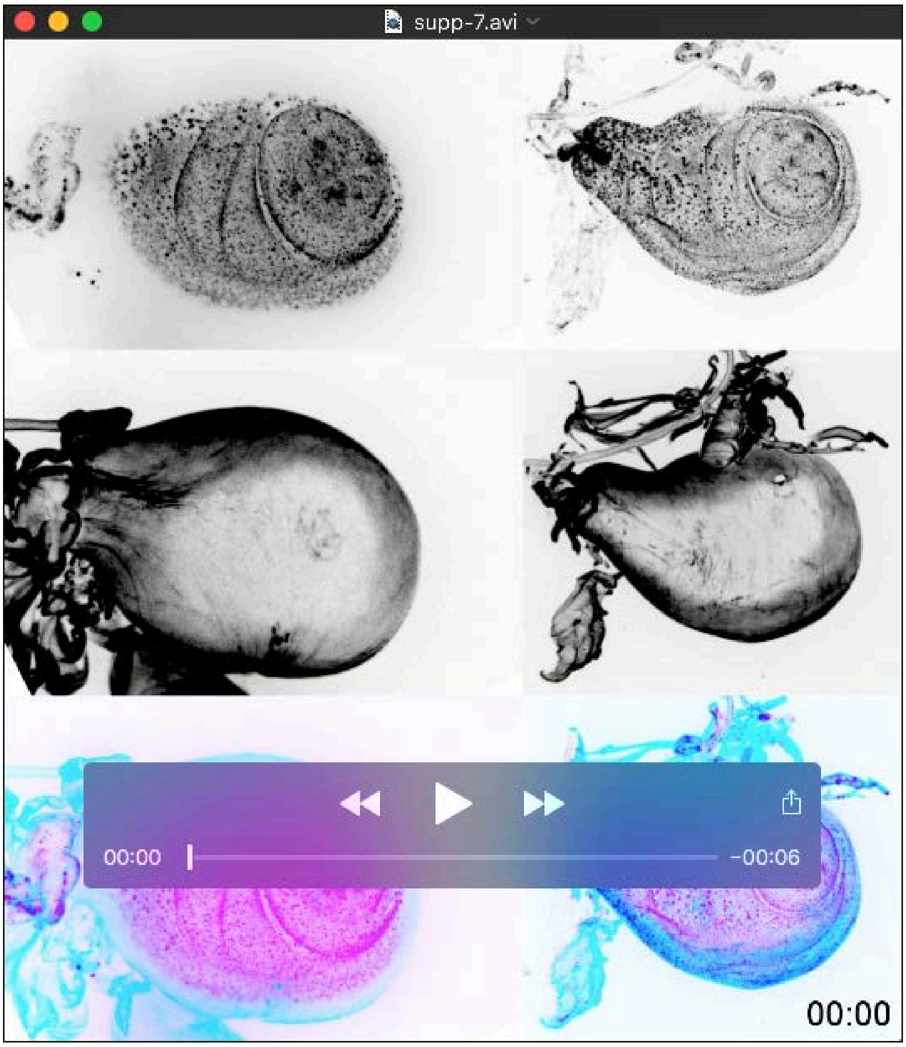

Movie 6. Related to Figure 5b and Figure S6. Matrix degradation is not required for PE opening.

Time-lapse confocal images (z-projections) of third-instar larval leg discs expressing fluorescent myosin (*sqh-TagRFPt[9B]*, top) and collagen (*vkg-GFP*, middle) and cultured with ecdysone to induce leg elongation. Bottom row: merged images (collagen: cyan, myosin: magenta). Left: control condition (0.5 % DMSO). Right: MMP inhibition (0.5% DMSO, 50 µM GM6001). Image stacks were acquired every 15 min. The ECM layer opens at the dorsal tip and retracts in the control condition, whereas MMP inhibition preserves ECM integrity at the dorsal tip. The PE cell monolayer opens and retracts in both conditions (control: n=7/7, GM6001: n=11/11).
